# Survival rates of homozygotic *Tp53* knockout rats as a tool for preclinical assessment of cancer prevention and treatment

**DOI:** 10.1186/s11658-017-0039-z

**Published:** 2017-05-18

**Authors:** Damian Strzemecki, Magdalena Guzowska, Paweł Grieb

**Affiliations:** Department of Experimental Pharmacology, Mossakowski Medical Research Centre, Polish Academy of Sciences, 5 Pawińskiego Str., Warsaw, 02-106 Poland

**Keywords:** *Tp53* knockout rats, Survival curve, Chemoprevention, Cancer

## Abstract

**Background:**

The gene that encodes tumor protein p53, *Tp53*, is mutated or silenced in most human cancers and is recognized as one of the most important cancer drivers. Homozygotic *Tp*53 knockout mice, which develop lethal cancers early in their lives, are already used in cancer prevention studies, and now *Tp*53 knockout rats have also been generated. This study assessed feasibility of using homozygous *Tp53* knockout rats to evaluate the possible outcome of cancer chemoprevention.

**Methods:**

A small colony of *Tp*53 knockout rats with a Wistar strain genetic background was initiated and maintained in the animal house at our institution. *Tp53* heterozygotic females were bred with *Tp53* homozygous knockout males to obtain a surplus of knockout homozygotes. To evaluate the reproducibility of their lifespan, 4 groups of *Tp53* homozygous knockout male rats born during consecutive quarters of the year were kept behind a sanitary barrier in a controlled environment until they reached a moribund state. Their individual lifespan data were used to construct quarterly survival curves.

**Results:**

The four consecutive quarterly survival curves were highly reproducible. They were combined into a single “master” curve for use as a reference in intervention studies. The average lifespan of untreated male *Tp53* homozygous knockout rats was normally distributed, with a median of 133 days. Sample size vs. effect calculations revealed that confirming a 20% and 30% increase in the lifespan would respectively require a sample size of 18 and 9 animals (when assessed using the *t*-test with a power of 80% and alpha set at 0.05). As an example, the *Tp53* homozygous knockout rat model was used to test the chemopreventive properties of carnosine, a dipeptide with suspected anticancer properties possibly involving modulation of the mTOR pathway. The result was negative.

**Conclusion:**

Further evaluation of the *Tp53* homozygous knockout male rat colony is required before it can be confirmed as a viable tool for assessing new methods of cancer prevention or treatment.

## Introduction

Potentially the best method to fight cancer could be chemoprevention, defined as the long-term intake of natural, synthetic or biological agents to reverse, suppress or prevent carcinogenesis in its initial phases or the progression of premalignant cells to invasive disease [1, 2]. A plethora of substances with different modes of action are under consideration as chemopreventives [3–7] and preclinical models to assess their efficacy would certainly be welcomed.

Since its discovery in 1979, *Tp53*, which encodes transcription factor p53, has become one of the most extensively studied factors involved in carcinogenesis. Mutations in *Tp53* are present in more than 50% of human malignancies [8]. Almost 30 years ago, *Tp53* knockout mice were used in experimental research on *Tp53*. These animals mature normally, but soon afterwards, all spontaneously develop lethal tumors. In null homozygotes (p53–/–), the average time to tumor development is 4.5 months, whereas half of the heterozygotes (p53+/–) develop tumors by 18 months [9].

The viability of *Tp53* null mice provided numerous opportunities to study cellular processes in the absence of wild-type *Tp53*. Using *Tp53* heterozygotes as a sensitive rodent model for testing known or suspected carcinogens has been advocated as it requires fewer animals and less time than conventional rodent carcinogenicity tests [10].

It has also been suggested that *Tp53* knockout homozygotic mice can be used to assess efficacy of cancer chemoprevention. In the first study of this type, it was found that imposing a caloric restriction on them to 60% of *ad libitum* consumption results in significant prolongation of survival, from a median of 16 weeks to 25 weeks [11]. In the next study, male p53(-/-) mice were treated with dehydroepiandrosterone (DHEA), quercetin, *d*-limonene, or *all*-*trans* retinoic acid. An average prolongation of survival by 58% was found only in the DHEA-treated group [12].

However, despite the authors’ conclusion that their results provided validation of *Tp53* knockout mice as a model for testing cancer chemoprevention strategies, only a few studies of this type have later been reported. In one [13], oral treatment with a synthetic DHEA analog (16a-fluoro-5-androsten-17-one) increased lifespan, similar to the effect of DHEA. In another [14], a prototypic mTOR inhibitor rapamycin supplied in drinking water extended the mean lifespan of p53+/- mice by 10 to 28%. The other two studies [15, 16] showed no prolongation of *Tp53* knockout mice survival upon pharmacological blockade of anti-apoptotic proteins belonging to the Bcl family or intake of a complex nutraceutical supplement containing ca. 35 ingredients.

*Tp53* knockout rats have been generated more recently. Tong et al. [17] disrupted the *Tp53* gene via homologous recombination in embryonic stem cells and showed germline transmission of the *Tp53* gene-targeted mutation. Van Boxtel et al. [18] used N-ethyl-N-nitrosourea-driven target-selected mutagenesis to obtain *Tp53* knockout rats and reported that both null *Tp53* homozygotes and *Tp53* heterozygotes start to develop lethal tumors, mainly sarcomas, at 4 and 8 months of age, respectively. McCoy et al. [19] created a *Tp53* knockout rat that contains an 11-bp deletion in exon 3, resulting in a frameshift and premature terminations in the open reading frame. The most recent report is that of Hansen et al. [20], who created a *Tp53* knockout rat based on the inbred rat strain Fisher-344. All these authors remarked that *Tp53* knockout rats could be highly suitable for oncology research, with van Boxtel et al. [18] concluding that *Tp53* knockout rats are highly complementary to existing mouse models for studying human cancer biology, and that due to early onset of spontaneous cancers, this model is unique and may be especially useful tool for testing therapeutic interventions.

Aggarwal et al. [21] noted that it is not clear whether genetically engineered mouse models in which tumor formation is driven by a clinically relevant oncogene or loss of a tumor suppressor gene (e.g., *Tp53*) can be better predictors of cancer drug discovery than experiments with cell cultures and models that involve non-genetically engineered animals. More recently, Prasad et al. [22] argued that in cancer-related drug discovery, serendipity and coincidence have thus far played a much larger role than rational drug discovery. Indeed, for several reasons, experiments with cell cultures and mice are poorly predictive of clinical results with new cancer treatments [23], and even less of successful chemoprevention [24]. However, *Tp53* knockout rats have not yet been used in such experiments.

In the animal house of our institution, we initiated a small colony of p53 knockout rats and maintained it over 1 year. Hereby we report on the reproducibility of the survival curves of male rat *Tp53* knockout homozygotes, and on their possible suitability as a tool for preclinical assessment of secondary cancer prevention.

## Methods

### Mating of animals

A colony of *Tp53* knockout rats (p53 TGEM Rat) with a Wistar strain genetic background was initiated and maintained in the animal house of our institution. Two pairs of male and female heterozygotic rats were purchased from Charles River under the exclusive license of Transposagen. They bear a single T to A point mutation in the *Tp53* DNA-binding domain that introduces a premature C to X stop codon in position 273aa [18]. These animals gave rise to our in-house population. To obtain a surplus of knockout homozygotes, heterozygotic parent crossing was to a great extent substituted with heterozygotic females crossing with homozygotic knockout males. Nevertheless, heterozygotic parent crossing is still being conducted to maintain a viable core population.

### Genotyping

The genotype of the *Tp53* knockout rats is determined using simple allele-discriminating PCR (SAP), as described by Bui and Liu [25], with a custom primer set based on *Rattus norvegicus* p53 mRNA sequence: NM_030989. The primer set is specific for the detection of a single nucleotide mutation in *Tp53*: *Tp53* mutant reverse primer (5′- GTCTCTCCCAGGACAGGTT -3′), *Tp53* wild-type reverse primer (5′-GTCTCTCCCAGGACAGGTA-3′), and *Tp53* common forward primer (5′-GAAGACTCCAGGTAGGAAGC-3′).

DNA is extracted from a tail snip using a GeneMATRIX EURx kit (EURx Ltd.) according to the manufacturer’s instructions. The tail biopsy is taken between 3 and 4 weeks of age. PCR is performed with an initial denaturation step at 94 °C for 6 min, followed by 35 cycles of 94 °C for 20 s, 52 °C for 20 s, 72 °C for 20 s, and final elongation step at 72 °C for 7 min.

### Survival curves of Tp53 knockout homozygotes

Because homozygotic knockout of *Tp53* appeared to be lethal for most of the female offspring, we restricted our experiment to homozygotic knockout male rats. To evaluate reproducibility of their survival, 4 groups of *Tp53* knockout homozygotic male rats born during consecutive quarters of the year were kept behind a sanitary barrier in the controlled environment of a pathogen-free animal facility. They had a 12-h light and dark cycle with the temperature maintained at 22 °C ± 1 °C and a relative humidity of 40–70%. Food and water were supplied *ad libitum*. When the animals reached moribund state, they were killed.

To avoid unnecessary pain and suffering, we applied predetermined criteria for evaluating the physiological and behavioral signs of disease. This approach enabled conscious interruption of the experiment (humane endpoint), while providing accurate and reliable data acquisition. Rats with observed changes in their physical appearance (rough coat, porphyrin exudate around eyes or nose) or behavior were examined with increasing intensity, namely by palpation of the whole body. Symptoms that required immediate discontinuation of the experiment (euthanasia of the animal) included: 1) weight loss from 15 to 20% within 7 days; 2) paralysis of limbs or neurological symptoms; 3) loss of ability to ambulate (inability to access food or water); 4) labored breathing; 5) tumor size exceeding 3 cm; 6) appearance of an open wounds, ulcers or tumor necrosis [26]. All the animal experiments were carried out in strict accordance with the guidelines of the IV Animal Ethical Committee in Warsaw (approval number 88/2015).

### Statistical analysis

All the calculations were performed using *R* Statistical Software. The statistical significance of disturbances in sex and genotype rates were assessed with chi-squared goodness-of-fit test (alpha 0.05). Individual lifespan data were used to calculate the Kaplan-Meier estimator and its estimated standard error (square root of the Greenwood formula), and the 95% confidence interval using the log transform. The normality of data distribution was checked with the Shapiro-Wilk normality test.

Power analysis for the two-group independent sample *t*-test was conducted to assess the number of animals necessary to obtain statistically significant data (alpha 0.05) for 3 preselected powers – 0.7, 0.8 and 0.9 – and expected effect size. The effect size describes the magnitude of a treatment effect, defined as the difference between the means, divided by the standard deviation of either group.

### Oral carnosine supplementation

The “field test” of the *Tp53* homozygous knockout rats as a tool for testing chemoprevention used L-carnosine, a naturally occurring dipeptide consisting of β-alanine and L-histidine. This compound has been suggested to influence cell proliferation, cell cycle arrest, apoptosis, and the glycolytic energy metabolism of certain tumor cells [27, 28]. It could be useful as a chemopreventive agent, but has not yet been tested in vivo for this purpose. In rats with streptozotocin-induced diabetes, chronic administration of L-carnosine in drinking water, 1 g/kg body weight per day, prevented the development of retinal vascular damage without producing appreciable toxic effects [29]. This suggests that such a dose of carnosine exerts significant physiological influence. To establish the dose of carnosine in terms of its content in drinking water, water consumption and body weight were assessed weekly. The appropriate dose of L-carnosine was then dissolved in the daily drinking volume, and replaced every 4 days. The treatment was initiated at 63 or 76 days of age.

## Results

### Viability and spontaneous cancers in p53 knockout homozygotes

We observed disturbances in the male-to-female ratio and the expected genotype ratio in both types of litter (after heterozygotic parent crossing and hetrozygotic female with knockout male crossing). However, the litter size seemed not to be affected, with average size of 9 ± 3 rats. Statistical analysis using the chi-squared goodness-of-fit test (alpha 0.05) showed that the observed reduction in the number of female pups in the litter (up to 40%) is significant, with *p* values of 0.002 and less than 0.001, respectively, for heterozygotic crossing and heterozygotic females and knockout male crossing litters. The same test (alpha 0.05) was used to evaluate the statistical significance of observed disturbances in the genotype ratios. In all the groups – females and males in heterozygotic crossing litters and females and males in heterozygotic female and knock out male crossing litters – the detected disturbance was statistically significant with the respective *p* values as follows: less than 0.001, 0.03, less than 0.001 and 0.05. We could observe a significantly reduced number of knockout pups, most probably due to a perturbation in embryo development, affecting mostly females (only 10% of the expected amount of female knockout pups were born). Moreover, the female knockout pups showed a reduced vitality.

Knockout rats developed grossly visible tumors and/or suffered marked conditional decline at 4 months of age at the latest. In our in-house population, we observed solid tumors and lymphomas. In the majority of the animals, between day 80 and 128 of life, clearly visible tumors (diameter over 0.3 cm) were detected under the skin in various locations (e.g., outer lateral surface of hind legs, armpit, chest) and the rats were euthanized (according to above described humane endpoint) from 9 to 36 days afterwards. We did not find any significant correlation between the day of tumor onset and the lifespan.

Our study aimed to assess the feasibility of using *Tp53* knockout rats for chemopreventive studies, so we did not histologically assess the tumors. Our general impression was that the most frequent tumor type (certainly more than half of all cases) appeared to be hemangiosarcomas present in different locations under the skin. Importantly, these tumors displayed high intratumoral hydrostatic pressure, resulting upon incision in an aggressive expulsion of the bloody fluid which filled the cyst. Also, approx. 10% of the animals had a noticeably low hematocrit.

### The reference survival curve

**Fig. 1:**
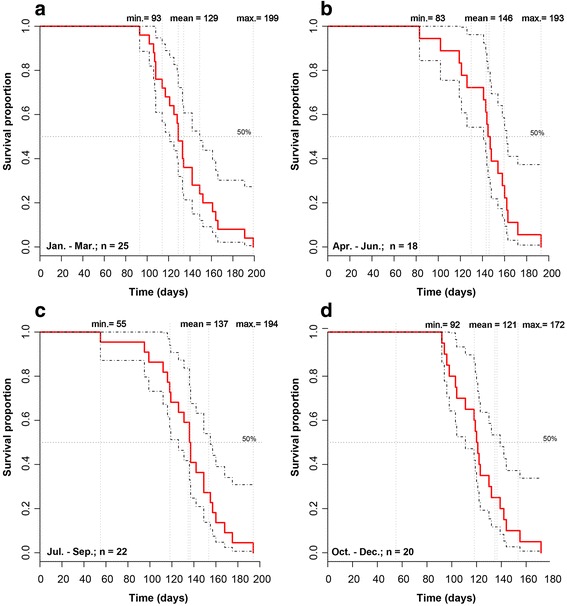
Comparison of survival curves illustrating the lack of significant influence of the season on the rats’ survival. A cohort of 85 *Tp53* homozygous knockout male rats was born during the full year. To assess if the seasons of the year have an influence on survival of rats, survival rates for each season were; **a** – winter: January 1–March 31, **b** – spring: April 1–June 30, **c** – summer: July 1–September 30, **d** – autumn: October 1–December 31

**Fig. 2:**
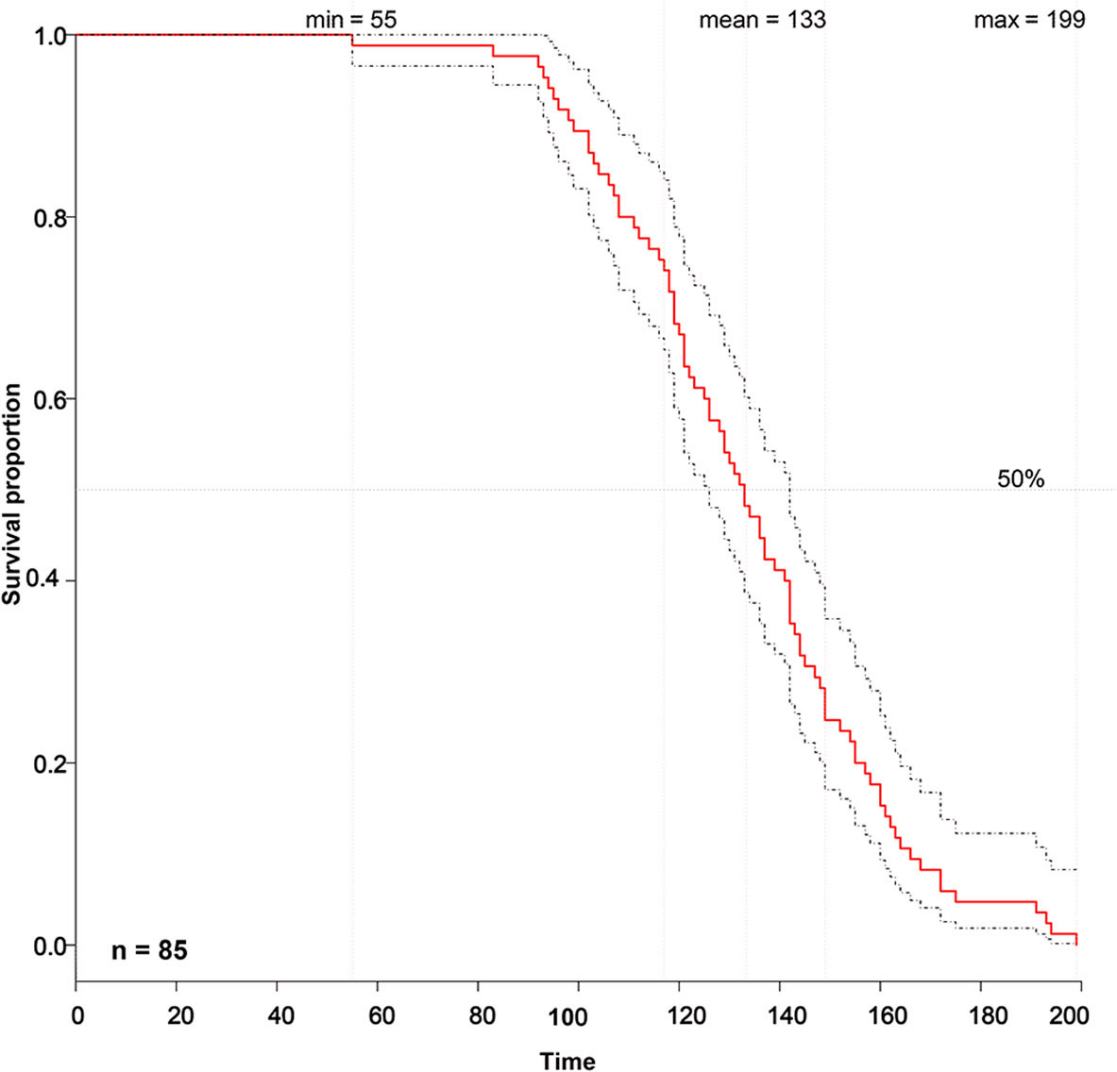
Kaplan-Meier survival curve constructed for a population of 85 homozygous *Tp53* knockout male rats. The survival data of each homozygous *Tp53* knockout male rat cohort were used to construct the K-M survival curve with CI = 95%, which can be further used as the historical reference survival curve

The 4 groups of knockout rats presented similar lifespan distributions (Fig. 1). This allowed a reference survival curve encompassing all the animals to be constructed. It can be used as historical control. The lifespan data were normally distributed (Shapiro-Wilk normality test *p* value = 0.7939), and the average lifespan was 133 days with a standard deviation of 27 days. The reference survival curve was constructed data for all 85 male p53 knockout rats (Fig. 2).

**Fig. 3:**
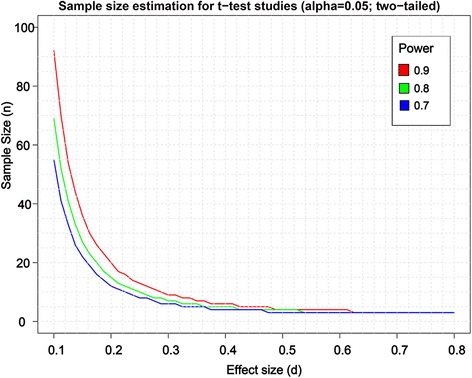
Sample size vs. effect size plot. The plot was constructed based on collected survival data for 3 different *t*-test powers (0.7, 0.8 and 0.9) and alpha = 0.05. The effect size (d) is the standardized difference between two means

According to the sample size (n) vs. effect size (d) calculations this model can, for example, detect a 20% effect with a sample size of 18 animals and a 30% effect with only 9 animals (for *t*-test with power of 80% and alpha set to 0.05) (Fig. 3).

### No effect of carnosine on survival

**Fig. 4:**
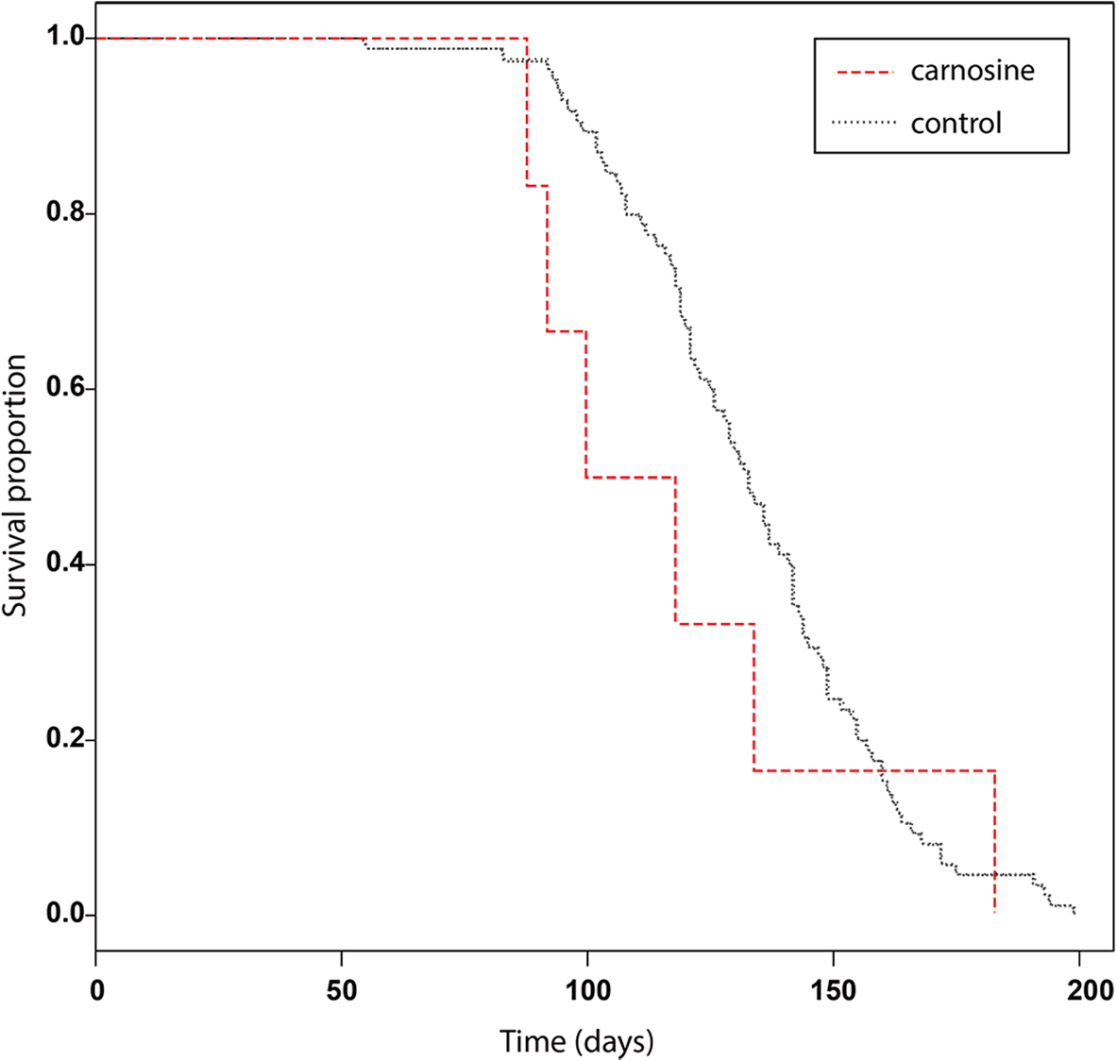
Kaplan-Meier survival curve after carnosine administration in comparison to the historical survival curve. The historical survival curve can be used as a reference curve in chemoprevention assessment experiments

To check the performance of our in-house rat population in chemoprevention assessment experiments, we administrated carnosine in water solution orally in an effective and nontoxic dose of 1 g/kg of body weight per 24 h. We did not find any sign of a positive effect of carnosine on survival (Fig. 4).

## Discussion

The homozygous *Tp53* knockout male rats used in this study were taken from a colony initiated with two pairs of heterozygotic rats purchased from Transposagen and should thus be identical to the animals described in the paper of van Boxtel et al. [18]. In that paper, the survival curves of homozygotic and heterozygotic knockouts were shown, and the median lifespans were 120 days and approx. 9 months, respectively. The genetic background for this model is Wistar strain, but similar lifespans were also reported for the homozygotic *Tp53* knockouts with a Sprague-Dawley strain (median of 126 days [20]) or Fischer 344 strain (median of 124 days [19]) background.

Although mice are the most popular experimental animal in many research areas (including experimental cancer research), some investigators favor rats because of their larger size, which facilitates experimental interventions, e.g., repetitive blood sampling or in vivo imaging studies with magnetic resonance or positron emission techniques [30]. The other important reason is that the metabolic physiology of the rat is considered closer to humans than that of the mouse, making the rat “inherently more translational” [31]. An example of rat cancer models being closer to human cancers than analogous murine models are rats with knockout or mutated *Apc* gene [32].

It is also worth mentioning that in humans with germline mutations of the *Tp53* gene leading to the Li-Fraumeni syndrome, sarcomas comprise approx. 95% of all tumors that occur before the age of 50 years [33]. Thus, the tumor spectrum in the *Tp53* homozygous knockout rats is similar to those in humans suffering from Li-Fraumeni syndrome, whereas *Tp53* knockout mice predominantly develop lethal lymphomas (although they develop sarcomas if development of lymphomas is genetically prevented) [34].

Both species (mouse and rat) have similar food intake expressed relative to body weight [35]. Since rats are approx. 10 times the size of mice, this makes experiments with rats significantly more expensive. Chemoprevention studies in rats may be *a priori* assumed to be prohibitively expensive. However, the stability and reproducibility of survival curves of *Tp53* male knockout rats may allow researchers to omit a synchronous untreated control, which will be helpful in curbing expenses. No large number of animals is required to become convinced that a compound does not display cancer-preventing properties in this testing system, as shown by the results of a field test of our approach.

We shall also note that hemangiosarcomas, which appear to be the most frequent type of tumor in *Tp53* homozygous knockout rats, may develop in rodents without involvement of mutations and DNA damage. Indeed, such tumors are well known to toxicologists, because they are induced by various industrial chemicals and pharmaceuticals. Interestingly, several of the hemangiosarcoma-inducing substances are non-DNA reactive, and the mode of action of some of them (namely 2-butoxyethanol, elmiron and *p*-chloroaniline) has been proposed to include hemolysis leading to iron overload [36]. As mentioned, in some 10% of our *Tp53* homozygotic knockouts, a very low hematocrit was evident. This serendipitous observation may reflect a hitherto unknown link between the *Tp53* gene and iron metabolism. A few years ago, it was suggested [37] that heme binding to Tp53 protein may establish a mechanistic link between iron overload and cancer development through promotion of its nuclear transport and cytosolic degradation. Such a mechanism would result in the downregulation of cellular p53 signaling during iron or heme excess, but it is obviously not acting in *Tp53* homozygous knockout rats. Some other explanation should be sought for the frequent development of anemia in these animals.

Carnosine, a naturally occurring dipeptide consisting of β-alanine and L-histidine and known primarily for its antioxidative properties, is nontoxic and readily available as a food supplement. We chose this compound because its inhibitory effects on neoplastic cell lines has been described, stimulating speculations on its possible usefulness in cancer treatment [38]. Moreover, it was recently suggested that one of the major targets of carnosine is the Akt/mTOR/p70S6K signaling cascade [39]. On the other hand, the mTOR pathway is modulated by caloric restriction and mediates its effects on lifespan extension [40]. Considering that caloric restriction has been shown to significantly increase the lifespan of homozygotic *Tp53* knockout mice [11], we reasoned that a positive effect of carnosine may occur in *Tp53* homozygous knockout rats.

As we have found, this is not the case. The most probable reason for carnosine failing to protect against cancer development in the *Tp53* homozygous knockout rats is that, in fact, the arguments for expecting anticancer effects of carnosine were poor. Although carnosine inhibited ATP production in primary cultures of human malignant gliomas and in established cultures of human gastric cancer, this effect occurred when the cancer cells were respectivel exposed to 50 mM and 20 mM concentrations of the peptide [41, 42]. These concentrations are one order of magnitude higher than the highest concentration of carnosine present in the rat body (in skeletal muscle, 2–6 mmol/kg wet tissue), several orders of magnitude higher than those in other tissues and body fluids [43], and higher than any that could ever be established in the blood of a living rat.

## Conclusions

Ma et al. [24] argued that many genetically edited and chemically-induced carcinogenesis models produce tumors that do not possess a full range of cancer features, and should therefore be used with caution in chemoprevention studies. It remains to be shown whether neoplasms that develop in the homozygotic *Tp53* knockout rats possess all or only some of these features. In any case, these animals may prove useful for evaluating a new type of anticancer drugs, proposed recently by Wang et al. [44]. Such new drugs should act differently than most currently used anticancer drugs, which are genotoxic and evoke apoptosis of cancer cells, but at the same time give them time to mutate and select resistant or metastatic clones. The *Tp53* knockout rats should also prove helpful for preclinical evaluation of gene therapy aimed at restoring the active *Tp53* gene in cancer cells in vivo. Preclinical tests of *Tp53* gene therapies have thus far been performed only in mice [45], and at least two such technologies are already available for clinical use, but only in China [46].

## Abbreviations

Akt: Serine/threonine kinase
Apc: Intestinal-cancer-associated adenomatous polyposis coli gene
ATP: Adenosine triphosphate
Bcl family: B-cell lymphoma regulator protein family
DHEA: Dehydroepiandrosterone
ENU: *N*-ethyl-*N*-nitrosourea
mTOR: Mechanistic target of rapamycin
p70S6K: Ribosomal protein S6 kinase
SAP: Simple allele-discriminating PCR
*TP53*: Human tumor protein p53
*Tp53*: Rat tumor protein p53
